# Visual cues that predict intuitive risk perception in the case of HIV

**DOI:** 10.1371/journal.pone.0211770

**Published:** 2019-02-20

**Authors:** Ralf Schmälzle, Freda-Marie Hartung, Alexander Barth, Martin A. Imhof, Alex Kenter, Britta Renner, Harald T. Schupp

**Affiliations:** 1 Department of Communication, Michigan State University, East Lansing, Michigan, United States of America; 2 Department of Psychology, University of Konstanz, Konstanz, Baden-Württemberg, Germany; 3 Department of Communication & Environment, Hochschule Rhein-Waal, Kamp-Lintfort, Nordrhein-Westfalen, Germany; West Virginia University, UNITED STATES

## Abstract

Field studies indicate that people may form impressions about potential partners’ HIV risk, yet lack insight into what underlies such intuitions. The present study examined which cues may give rise to the perception of riskiness. Towards this end, portrait pictures of persons that are representative of the kinds of images found on social media were evaluated by independent raters on two sets of data: First, sixty visible cues deemed relevant to person perception, and second, perceived HIV risk and trustworthiness, health, and attractiveness. Here, we report correlations between cues and perceived HIV risk, exposing cue-criterion associations that may be used to infer intuitively HIV risk. Second, we trained a multiple cue-based model to forecast perceived HIV risk through cross-validated predictive modelling. Trained models accurately predicted how ‘risky’ a person was perceived (*r* = 0.75) in a novel sample of portraits. Findings are discussed with respect to HIV risk stereotypes and implications regarding how to foster effective protective behaviors.

## Introduction

Merely looking at another person, people spontaneously form impressions about fundamental personality characteristics such as trustworthiness, competence, and attractiveness [1–4]. Such snap judgments have been shown to influence real-world decisions in many contexts, including political voting, sentencing, leadership, or online dating [5–9]. Less known, however, snap judgments seem also to play a role in the context of sexually transmitted infections (STIs) [10,11] where people draw inferences about potential partners and make decisions about whether to use effective protection.

Research shows that people report that they often 'just know' whether a person is risky or safe—even when they do not know much about the respective person's past sexual behavior or personality [12,13]. For example, many people who contracted HIV during unprotected sexual intercourse report they had assumed their partners were safe–and that they regret being wrong [14,15]. Focus groups on HIV prevention point to a related phenomenon: participants often express trust in their ability to detect potentially risky sex partners based on their appearance, even though research suggests that they cannot [16,17]. Impressions of partner safety or risk based on intuitive snap judgments may thus influence reliance on effective protection strategies (i.e., condom use), particularly during ‘hot’ sexual encounters that impair deliberation and make impulsive decisions more likely [18–21].

Intuitions about riskiness may not only influence sexual risk behavior, but may also come into play in the medical context, for instance, when paramedics or nurses approach patients or victims and need to decide on the spot whether or not to wear protective gloves (see [22] for a similar argument). Furthermore, a recent study indicated that a mismatch between patients’ personal profile and the stereotypical risk factors for HIV resulted in delayed diagnosis of HIV by doctors [23]. Not only did the participants report surprise at their diagnosis, indicating that people without the stereotypical risk factors for HIV fail to make the connection between their behaviors and risk of HIV, but medical experts made the same error: The participants reported having seldom been offered HIV tests despite visiting physicians over a period of years with HIV symptoms, i.e., weight loss, persistent infections, swollen lymph nodes, suggesting that the stereotypes of who is at risk of contracting HIV are also present in the medical community.

The intuitive nature of risk perception has been strongly supported by a series of studies relying on neuroscientific measures. Event-related potential (ERP) studies revealed that ERP responses to risky as compared to safe individuals diverged early in the processing stream (< 300 ms). This speed precludes systematic reasoning about health risk and thus supports the notion of intuitive processing [24]. Moreover, ERP differences between intuitively risky and safe partners emerged at the level of the late positive potential (LPP), an ERP component known to respond to affective significance [25]. Portraits of risky-looking individuals prompted larger LPPs, which may serve as an intuitive alarm signal for attentive processing [26–28]. This interpretation was corroborated by a subsequent fMRI study that found increased activation toward individuals later judged as risky within the saliency network, a set of brain regions involved in attention and relevance detection [29]. Perhaps the strongest support for the intuitive nature of HIV risk perception comes from studies which revealed similar ERP and fMRI correlates of risk processing for implicit and explicit conditions [28,29]. Together, these findings indicate that people are highly sensitive to cues of riskiness and rapidly and spontaneously form impressions about HIV risk.

To tap into the nature of the associative memory representation underlying HIV risk stereotype, several studies related HIV risk perceptions to a broader set of person characteristics. Across different stimulus sets, a strong inverse association of HIV risk perception with ratings of trustworthiness and responsibility emerged [27,30]. This is consistent with work suggesting that trustworthiness and responsibility lie at the core of a high HIV risk stereotype in young adults [31]. Interestingly, and compatible with the ERP findings, research on person perception indicates that inferences about trustworthiness are formed spontaneously and based on the information available in short glances [32,33]. For example, judgements of traits like trustworthiness or health state have been linked to facial features, shape and color [3,34]. These findings show that humans form impressions—whether about risk or trustworthiness—with ease, but they do not imply that the inferences are reliable or accurate. Additionally, both commonalities and differences have been found for cue-based judgements across cultural backgrounds, highlighting the importance of possible cross-cultural differences [35,36]. In sum, an extant literature in evolutionary psychology and face perception has identified that such cues matter in a wide array of judgements, such as trustworthiness, attractiveness, and health [35,37–39]. Furthermore, there is a robust literature in social psychology regarding personality impressions based on thin slices of information [40], part of which also focuses on underlying cues [41–43].

**Fig 1:**
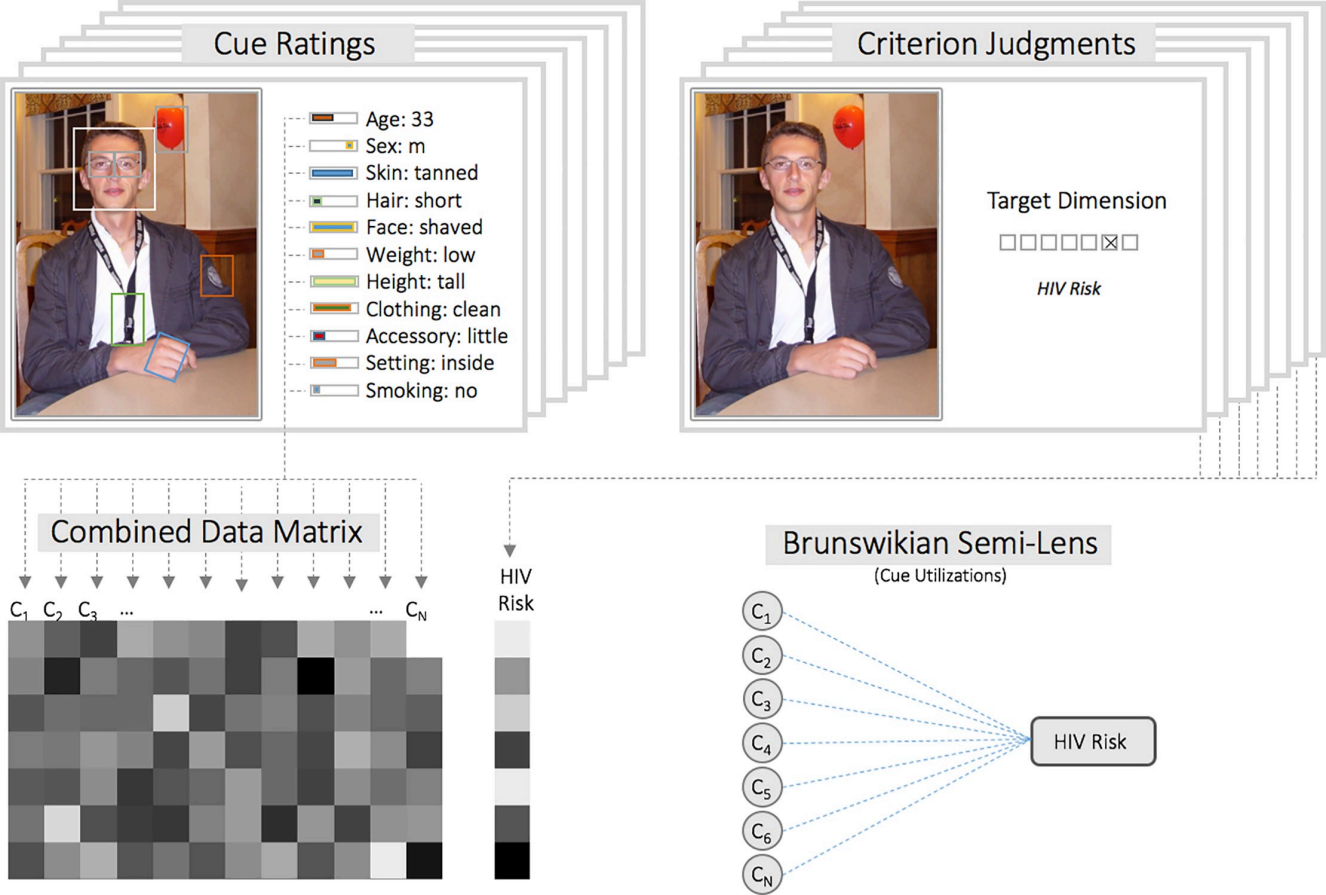
Schematic overview of the current study and example stimuli. Ratings for observable cues and criterion ratings (HIV riskiness, as well as other impressions) are collected from independent groups of raters for a large set of target photographs. Averaged cue and criterion ratings are then combined and correlations are assessed between each individual cue vector and the HIV risk criterion judgments. This strategy identifies cues that may be utilized to infer HIV risk and thus comprise a ‘Brunswikian Semi-Lens’.

The current study examined this issue using portraits that were similar to how people present themselves on social media and dating websites, creating ecologically valid conditions for the study of HIV risk impressions. Thus, our research focuses on early stages of HIV infection which are not reliably associated with visible signs of illness or health deterioration. The research design consisted of two independent sources of rating data: First, the entire stimulus set was evaluated according to a list of specific cues deemed relevant to person perception. Towards this end, a list of perceivable cues was derived from the literature and via focus groups, resulting in a set of over sixty cues and general impressions (see Table 1 and Fig 1). Each portrait was then rated on all cues to measure the extent that a cue was present. Second, an independent group of raters evaluated the depicted persons according to HIV risk as well as further person characteristics of trustworthiness, health, and attractiveness. These additional characteristics were assessed to determine the relationship of HIV risk perception to fundamental person characteristics brought out by previous research on trust [3,4,44], health [34,39], and attractiveness [36,45,46].

**Table 1:** Descriptives for cue ratings, correlations between cues and perceived HIV risk (‘cue utilization coefficients’), and regression model coefficients.

Cue	HIV Risk	* *	* *
Face: Eyes	*Mean (SD)*	*r*_*Cue-HIV Risk*_	coef_Lasso_
Dark (vs. no dark rings under eyes)	3.27 (0.87)	0.21	0.04
Reddened (vs. no reddened eyes)	2.85 (0.78)	0.33	0.12
Dark (vs. bright eyes)	4.3 (1.28)	0.23	0
**Face: View**			
Coquettish (vs. no coquettish gaze)	3.49 (1.22)	0.13	0.12
Averted (vs. front facing gaze)	3.42 (2.06)	0.14	0.01
Tired (vs. alert gaze)	3.45 (1.03)	0.28	0
**Face: Hair**			
Ungroomed (vs. groomed hair)	3.34 (0.87)	0.19	0.04
Long (vs. short hair)	3.31 (1.33)	0.19	0
Fashionable (vs. unfashionable hairstyle)	3.64 (1.03)	0.06	0
Dark (vs. bright hair)	4.63 (1.59)	0.22	0.03
**Face: Mouth**			
Smile (vs. no smile)	3.62 (1.56)	-0.33	0
Full (vs. narrow lips)	3.8 (0.93)	0.29	0.07
**Face: Skin**			
Unhealthy (vs. healthy skin)	3.41 (0.9)	0.14	0
Pale (vs. tanned skin)	4.24 (0.94)	-0.06	0
Pimply (vs. pimple free skin)	3.02 (0.84)	0.1	0
Many (vs. few skin folds)	2.49 (0.68)	-0.05	0
Spotty (vs. spot free skin)	2.74 (0.89)	0.1	0
Greasy (vs. dry skin)	4.31 (0.71)	-0.01	0.02
Lots of (vs. no skin visible)	4.65 (1.36)	-0.1	0
**Facial Configuration**			
Babyish (vs. mature face)	4.01 (0.97)	-0.09	0
Feminine (vs. masculine face)	4.16 (1.09)	-0.06	-0.03
Round (vs. narrow face)	3.6 (1.09)	-0.25	-0.01
Worn (vs. fresh face)	3.53 (0.96)	0.33	0.2
Ugly (vs. beautiful face)	3.93 (1.05)	0.01	0.02
Red (vs. pale cheeks)	4.03 (1.06)	-0.13	0
Narrow (vs. full jaws)	3.81 (1.19)	0.26	0.02
Average (vs. unusual face)	4.33 (0.77)	-0.42	-0.08
Reddened (vs. no reddened face)	3.93 (1.38)	-0.09	0.01
Symmetric (vs. unsymmetric face)	4.37 (0.77)	-0.01	0
**Facial Expression**	** **	*** ***	** **
Happy (vs. sad expression)	4.43 (1.5)	-0.33	0
Exhausted (vs. powerful expression)	3.7 (0.95)	0.24	0
Worried (vs. unworried expression)	3.26 (1.19)	0.31	0.06
Serious (vs. blithely expression)	3.72 (1.37)	0.35	0
Angry (vs. cheerful expression)	3.4 (1.08)	0.22	0
Friendly (vs. grumpy expression)	4.34 (1.39)	-0.35	-0.03
**Body: Figure**			
Musculous (vs. not musculous stature)	4.18 (0.89)	-0.09	-0.04
Overweight (vs. underweight)	3.7 (0.93)	-0.28	-0.11
Well (vs. badly proportioned stature)	4.69 (1.09)	0.23	0.07
Tall (vs. low height)	4.33 (0.81)	-0.13	-0.09
Tense (vs. relaxed posture)	3.76 (0.91)	0.16	0
**Body: Appearance**			
Ungroomed (vs. groomed appearance)	3.22 (1.04)	0.18	0.02
Lot of (vs. no body adornment)	2.96 (1.11)	0.43	0.14
Worn out (vs. intact clothes)	2.45 (0.81)	0.07	0
Provocative (vs. reserved clothes)	4.09 (1.23)	0.23	0.06
Unconventional (vs. conventional appearance)	3.49 (0.74)	0.54	0.31
Fashionable (vs. unfashionable appearance)	4.25 (1.08)	0.2	0.06
Dark (vs. bright clothes)	4.08 (1.6)	0.06	0.03
Clean (vs. dirty clothes)	4.38 (1.02)	-0.03	0
**Setting**			
Pallid (vs. colorful background)	3.96 (1.41)	0.15	0.04
Unorganized (vs. organized background)	3.74 (1.14)	0.07	0.05
Alcohol (vs. no alcohol visible)	0.12 (0.29)	0.09	0
Picture taken inside (vs. outside.)	7.47 (0.46)	0.08	0
Picture taken in nature (vs. civilization.)	0.25 (0.37)	-0.18	0
Cigarettes (vs. no cigarettes visible)	7.05 (0.15)	0.17	0
Food (vs. no food visible)	7.14 (0.27)	-0.08	0
During sports activities (vs. not.)	5.91 (2.4)	-0.06	-0.02

We first report the intraclass-correlation to support the notion that perceived riskiness is reliably measured and shared among participants. Secondly, we adopted a Brunswikian Lens Model perspective to study the relationship between perceivable cues and HIV riskiness ratings [47]. In brief, Brunswik’s Lens Model serves as framework for the relationship between the environment and perception, which is mediated probabilistically by cues that are used to form impressions [34,41,42,47,48]. Critically, we do not consider the relationship between cues and the objective external variable, i.e. ‘actual HIV status’, called cue-validity coefficients, but rather focus on the psychological impression of ‘perceived HIV risk’ or intuitive riskiness, i.e. the *cue-utilization* coefficients (see Fig 1). Next, we test whether a model based on multiple cues can be trained to predict HIV risk impressions in new data. Lastly, to reveal the relationship between cue-based models of HIV risk and models of trustworthiness, and their distinctiveness to models of attractiveness, we test whether cue-models trained to predict one variable can predict another (e.g. whether a cue-model that accurately predicts trust can be used to predict risk, and vice versa).

## Method

### Participants

The cue-ratings of 60 cues and 13 additional general impressions (cf. Tables 1 & 2) were collected within small groups of 8–12 participants aged between 18 and 35 in the context of an introductory psychology practicum. Participants received course credit whether they participated or not and provided oral consent as this was an anonymous survey. The criterion ratings of perceived HIV risk were obtained from previous studies of HIV risk perception, which accumulated criterion ratings across several separate studies [27–30]. For each photograph criterion ratings of HIV riskiness were obtained from 40 opposite-sex judges, who were between 20 and 32 years old. Participants were recruited for a study of first impressions on the local campus and participated in individual sessions. Criterion raters provided written consent to take part in the study and received either credit towards research participation requirements or monetary reimbursement. The research design and consent approach was approved by the IRB of the University of Konstanz. All procedures were in accordance with local guidelines and the principles expressed in the Declaration of Helsinki.

**Table 2:** Descriptives for ratings of general impressions.

General Impression	*Mean (SD)*	*r*_*Cue-HIV Risk*_
Irresponsible (vs. responsible)	3.6 (1.02)	0.6
Uneducated (vs. educated)	3.55 (0.9)	0.55
Selfish (vs. unselfish)	4.06 (1.1)	0.48
Ill-looking (vs. healthy-looking)	3.59 (1.23)	0.18
Scruffy (vs. kempt)	3.7 (1.1)	0.17
Southern (vs. nordic) type	3.31 (0.98)	0.34
Homosexual (vs. heterosexual)	0.15 (0.17)	0.13
Attractive (vs. unattractive)	3.93 (1.33)	0.1
Sporty (vs. unsporty)	4.39 (1.19)	0.04
Self-confident (vs. not)	4.6 (1.04)	-0.01
Popular (vs. unpopular)	4.07 (1)	-0.16
Cautious (vs. risk-seeking)	4.01 (1.03)	-0.36
Likeable (vs. unsympathetic)	4.28 (0.96)	-0.41

The middle column shows, for each impression, the average impression rating and the standard deviation. The right column lists the relationship between each general impression and perceived HIV risk.

### Cue judgment methods

#### Selection of cues

Each photograph was evaluated according to general personality characteristics and a large set of perceivable cues. General personality impressions are listed in Table 2 and were selected based on previous research on person perception [41–43,48,49]. In order to implement a Brunswik’s Lens Model perspective, it is crucial to represent the possible features and characteristics underlying snap judgments in the list of cues. Towards this end, in a first step, a large set of possible cues and impressions was identified by an extensive literature search of the relevant literatures on personality impressions and person perception [48,50, 51], which included journals from social and personality psychology, face perception and vision, health psychology as well as human medicine textbooks. From these diverse sources, we compiled a large list of several hundred possible cues, and conduced a focus group consisting of the two lead-Postdocs, two doctoral students and several research assistants to: (i) Identify additional potential cues for HIV risk impressions, that were not listed in the literature (“identify the white space”). (ii) Classify the cues across the spectrum from concrete (e.g. “cigarette visible”)—semi-abstracted (e.g. “smile”),—abstracted (e.g. “trustworthy’). (iii) Form broader categories along which the cues can be clustered (e.g. facial cues, adornment, setting). (iv) Reduce redundant or synonymous cues (e.g. “torn clothing” instead of torn shirt and torn shorts)” and (v) Remove cues that were not possible to rate with the present stimulus set (e.g. cues that are not visible, such as skin conditions of covered body-parts). The final list consisted of 60 perceivable cues which were sorted into ten higher order categories of face and body appearance and setting (see Table 1) as well as 13 general impressions (see Table 2).

#### Stimuli

The stimulus set consisted of 240 photographs of persons in daily life scenes and was the same as in previous research [27,28,52,53]. To have high ecological validity, stimuli were selected based on the following six criteria: (i) A colored photo of a (ii) single person located in the foreground, with (iii) their face clearly visible. In terms of age and race, only photographs of (iv) young (18–35 years old) (v) European descent were included. To resemble natural conditions and to facilitate impression formation, only (vi) portraits of individuals in which context features beyond the face itself were visible, such as attire, clothing, or aspects of the situation in which the picture was taken. Half of the pictures depicted male and female targets, respectively. The photographs were retrieved with permission from a popular online photo-sharing community (www.flickr.com).

#### Cue rating procedure

Booklets for collecting ratings were distributed across the groups with 8–10 cues or general impression rating scales for each participant. The study was conducted by an experimenter who presented 60 target photographs on a projector screen and participants provided their ratings for a given cue in booklets. Each participant rated all 60 photographs on one cue and then viewed them again with the instruction to rate the next cue. With this procedure, which lasted about 45 minutes per group, we obtained cue-ratings from eight judges per cue [54]. The study was presented as a general inquiry of person perceptions. Specifically, participants did not know that cue-ratings would be related to criterion judgments of HIV risk or other personality characteristics.

### Criterion judgment methods: HIV risk

#### Stimuli and procedure

Criterion ratings were obtained for the same stimulus set as in the cue rating study. These ratings were obtained by aggregating data from previous studies in order to obtain a ratio of 40 criterion judgments per image from opposite-sex raters [27,28]. Each participant was tested individually and the rating procedure was operated by a computer running Presentation software (Neurobehavioral Systems Inc., Berkeley, CA). Each picture was shown for 2 s followed by the presentation of the rating scales. The order of rating scales was randomized for each image, and the order of the picture stimuli was determined randomly for each participant. Perceived HIV risk was assessed by the question ‘How likely do you think is it that this person is HIV-positive?’ (translated from the German "Für wie wahrscheinlich halten Sie es, dass diese Person HIV-positiv ist?") on a 7-point likelihood rating scale ranging from ‘very unlikely' [1] to ‘very likely’ [7]. In addition to perceived HIV risk, participants also provided ratings of perceived trustworthiness, health, and attractiveness for each image [27,45]. In order to determine the relationship of visual cues and risk perception, it is necessary to demonstrate that the stimulus materials actually varied in their ascribed HIV risk. Thus, risk ratings were calculated across participants for each individual picture. Indicating substantial variation in risk, mean risk ratings increased linearly from very low risk (*min* = 1.83) to very high risk (*max* = 5.93; *M* = 3.7; *SD* = 0.86). Similar findings emerged for trustworthiness (*min* = 1.63; *max* = 6.25; *M* = 4.1; *SD* = 0.82), attractiveness (*min* = 1.35; *max* = 6.45; *M* = 3.67; *SD* = 1.16), and health (*min* = 2.12; *max* = 6.3; *M* = 4.4; *SD* = 0.83).

### Data analysis

We first examined the reliabilities of aggregate ratings and compute associations between individual cues and the criterion ratings of HIV risk. Next, we combined cues into a multiple regression model. Specifically, we used regularized LASSO regression with 10-fold cross-validation as implemented in scikit-learn [55,56]. Thus, within each fold, a training model of cue-criterion relationships was constructed based on a subset of the data. To validate the model, the coefficients from the training model were then applied to the held-out set of photographs that were not used for training. In other words, multiplying the cue-values for novel photographs with the trained model coefficients, the model generates cue-based predictions of HIV risk. These are then compared against the actual HIV risk ratings (see Fig 1) and predictive accuracy is measured using R^2^ and the standard error of the estimate, and averaging them across folds. Code to reproduce these analyses is posted online at http://github.com/nomcomm/ RiskCues_PlosOne.

## Results

### Reliability measures

We first assessed the reliabilities of the cue-ratings among the eight raters who evaluated to what extent each cue was expressed on the images (intraclass correlations, two-way random, absolute). Across the set of cues, the average ICC was .84 (see S1 Table for results for individual cues). As for the cues, we assessed the reliability for the criterion ratings. HIV risk had an ICC of .92 and similar values were obtained for ratings of trust, health, and attractiveness (all ICC = .91, see S1 Table).

### Relationships between individual cues and perceptions of HIV risk

The cue-utilization correlations in Table 1 indicate the relationships between each cue and perceptions of HIV risk. As can be seen, multiple cues, mostly showing low-to-moderate effect sizes, are related to perceived riskiness/safeness. For instance, if a person wears lots of body adornment, has an unconventional appearance, or appears tired-looking, then independent judges will regard this person as having higher HIV risk. Furthermore, the visibility of cigarettes, provocative clothing, and a facial expression signaling negative emotional states (angry, exhausted, serious, or worried) are also linked to heightened HIV risk. Conversely, lower HIV risk—or a safer impression—is associated with having positive emotional expressions (smiling, friendly, or happy), an average face, and with persons who are observed within nature scenery.

### Predicting HIV risk impressions from visual cues

**Fig 2:**
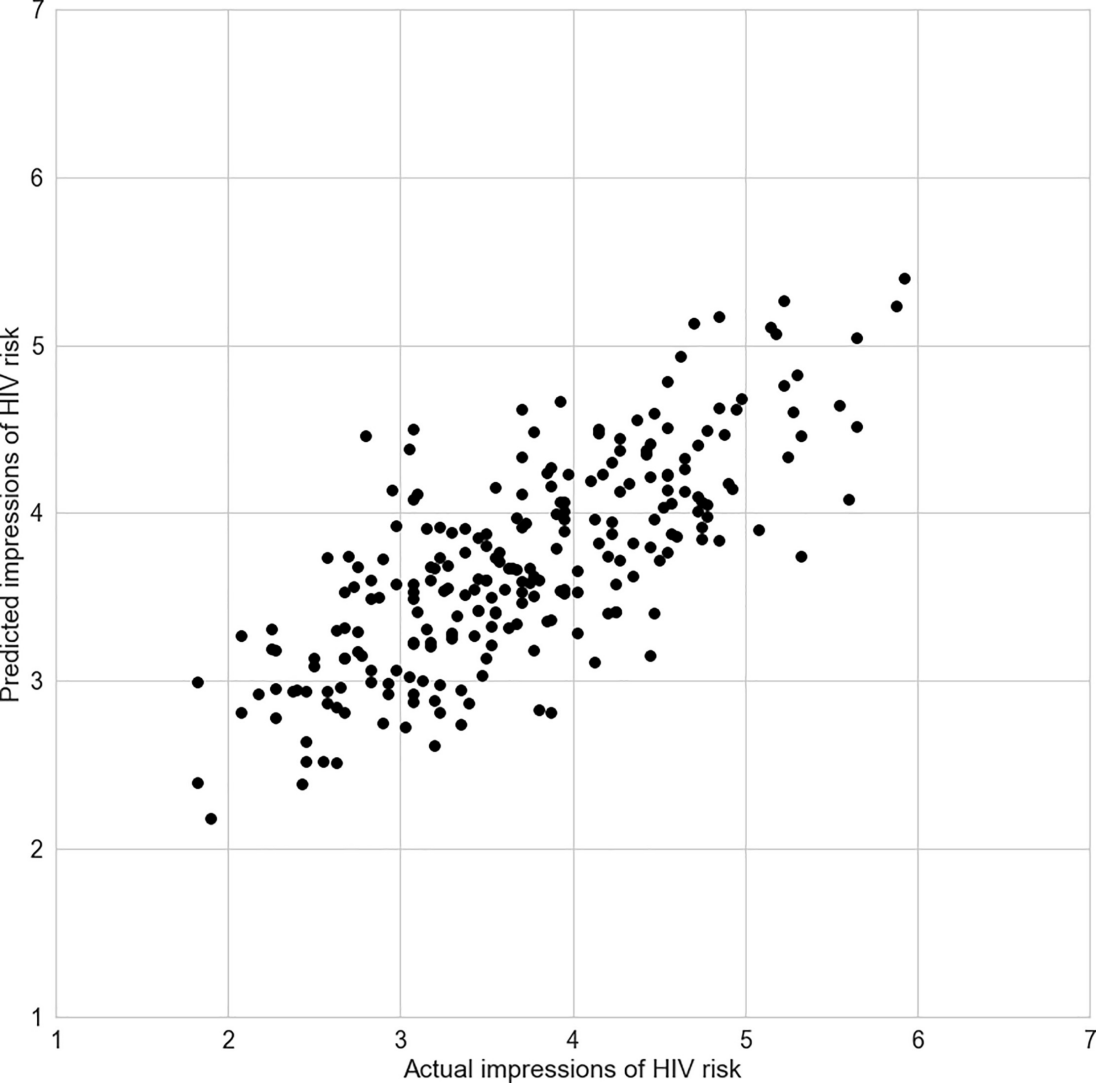
Relationship between actual impressions of HIV risk and model-based predictions. The model was trained using LASSO-Regression and cross-validated using a 10-fold strategy. We then compare the model-based predictions against actual perceptions of HIV risk obtained from different raters, finding that the model learned to successfully predict HIV risk based on cues (r = 0.75). See text for details.

We used a linear regression model to capture the relationship between cue-ratings and HIV impressions and to test whether and to what degree a trained model could predict HIV risk ratings in unseen data based on the learned cue-criterion relationships. Specifically, we used regularized LASSO regression with 10-fold cross-validation as implemented in scikit-learn [55–57]. We found that the model performed substantially above chance, explaining on average more than half of the variance, with an *r*^*2*^ = 0.56 and a *standard error of the estimate* = 0.57. Fig 2 shows the correlation between predicted and actual HIV risk ratings, which amounts to *r* = 0.75 (*t(238)* = 17.5, *p* < 0.0001). Thus, a model trained to predict HIV risk solely from visual cues can accurately predict HIV impressions for new pictures. Notably, even when confining the set of predictive cues to concrete ones (e.g. dark vs. bright clothes) and disregarding more abstract inferences (such as ‘conventional vs. unconventional appearance’), the predictive model still performed remarkably well (*r* = 0.61; *t(238)* = 11.9, p < 0.0001, *r*^*2*^ = 0.35, *standard error of the estimate* = 0.69). Also, applying conventional linear instead of LASSO regularized regression did not meaningfully change these findings. Together, these findings suggest that intuitive inferences about HIV risk—rather than being unknowable and random—are systematically related to visual cues.

### How does HIV risk perception relate to general person impressions?

Impressions about HIV risk are likely embedded in a network of various other social dimensions that might also be inferred from short glances [27,49]. To gain insight into these relationships, we also obtained ratings for all images on 13 more general impressions. Table 2 lists correlations between each general impression and perceived HIV risk. As shown, perceived low responsibility is most strongly associated with perceived HIV risk. This is consistent with previous research on HIV risk [27,31] and with recent work on social impressions [58], where this dimension also ranks first for impressions of trustworthiness. Further, risky-looking people are seen as rather uneducated and selfish, and as less cautious, less likeable, and less popular.

### The relationship between impressions of HIV risk to perceptions of trust, health, and attractiveness

The same methods used to examine and forecast ratings of HIV risk can also be applied to other criterion ratings, such as trust, health, and attractiveness. Specifically, the correlation between predicted trust and actual trust ratings was *r* = 0.75, for health *r* = 0.84, and *r* = 0.89 for attractiveness (all *p < 0*.*001)*. This suggests that for each of these characteristics, the overall impression of a given person can be predicted with relatively high accuracy from a set of cues. Importantly, the trained model based on trust was able to significantly predict HIV risk ratings, with lower trust ratings being associated with higher HIV risk (*r* = -0.64, *p* < 0.001). In contrast, the health and attractiveness models did not predict ratings of HIV risk very well, *r* = -0.23 for health and *r* = 0.06 for attractiveness.

## Discussion

Sexually transmitted infections are a great burden at the individual and societal level, with more than 1 million of STIs being acquired per day [59,60]. Major health organizations consistently report and warn against a knowledge-behavior gap in HIV prevention. Specifically, while knowledge about effective HIV prevention is high, levels of protective behavior are low [60]. The reliance on ineffective rather than effective strategies to prevent infection may help to explain the knowledge-behavior gap [17]. Specifically, basing HIV perception on the appearance or trustworthiness of the partner may give people the feeling of risk control while not providing effective control. In order to further understand appearance-based HIV risk perception, we reveal in the present research how visible cues in photographs of persons—similar to the one’s used in online dating—relate to impressions of HIV risk, how impressions of HIV risk can be predicted from the cues alone with relatively high accuracy, and how impressions of HIV risk relate to broader impressions of trust, health, and attractiveness.

Perhaps the most notable finding of this study is that ‘how risky’ a novel person will be evaluated on average can be predicted by their appearance. By linking the cue-ratings to the criterion judgments through cross-validated predictive modeling, the trained model significantly predicted HIV risk ratings for new target photographs. Importantly, all that is ‘fed in’ to this model are the cue-measures for each image, which are then weighed by the learned coefficients to yield predictions of HIV risk. In particular, we found that cue-based models can explain about half of the variance in perceived HIV risk, and that this finding is robust to specific modeling choices. These numbers are surprisingly high considering that (1) cues and criterion reflect consensus ratings not considering individual variation in appearance-based risk perception, (2) measurement errors in the assessment of cues and criterion, and (3) that additional relevant cues may not have been incorporated in the model. Inspection of the model coefficients revealed the most important cues for riskiness or safety. The top five risk-enhancing cues were ‘unconventional appearance’, a ‘worn face’, ‘lots of body adornment’, a ‘coquettish gaze’ and ‘reddened eyes’. In contrast, cues associated with more safety were a ‘friendly expression’, a ‘musculous stature’, an ‘average face’, a ‘tall body’ and ‘overweight’. Of note, as some of these cues are themselves abstractions (e.g. ‘unconventional appearance’), we also inspected top-ranking cues in the models that were reduced to concrete-only cues. In that model, additional top cues for HIV risk were ‘alcohol visible’ and ‘spotty skin’, whereas lowered risk was predicted by ‘picture taken in nature’, ‘food visible’ and ‘pimply skin’. Overall, when it comes to judgments of HIV risks at zero acquaintance, information contained in a fairly small set of visible cues is systematically related to the consensus among perceivers about what constitutes ‘HIV riskiness’.

The present findings close a gap of knowledge in the understanding of the processes leading to intuitive decisions to engage in risky sexual behaviors. We proposed previously that riskiness is judged according to a stereotype of HIV [27,28]. This reasoning builds upon the findings that distrust and lack of responsibility are key features of a high HIV risk stereotype [61], that HIV risk ratings are negatively associated with ratings of trust and responsibility [27,28], and evidence that trust is perceived intuitively [1]. According to this reasoning, HIV risk and trustworthiness are assumed to rely on common cues. The observed cue-utilization coefficients strongly support this notion. Specifically, a model trained on cues for trustworthiness was able to significantly predict lowered perceptions of HIV risk, and the model trained on HIV was able to predict perceived trustworthiness. Further insight into the nature of the suggested HIV risk stereotype can be gleaned by inspecting the relationship between HIV risk and ratings of 13 other general impressions. The strongest association emerged between HIV risk and a perceived lack of responsibility, followed by the perception that the depicted person was uneducated, selfish, and generally less likeable (see Table 2). This replicates previous findings obtained using verbal procedures to elicit stereotypic associations [27,61]. Overall, adopting a Brunswikian Lens Model perspective supports the notion that intuitive HIV risk perception may reflect the activation of the high HIV risk stereotype utilizing cues associated with trust and responsibility.

While the present study specifically focused on the cue-utilization process, it has to be noted that the cues utilized in intuitive risk perception are presumably not valid. To our knowledge, only one study directly examined the ability to detect early stage HIV positive individuals by presenting pictures and short vignettes from HIV-positive and HIV-negative people [17]. Participants were at chance level in detecting HIV risk, which suggests that relying on snap judgments of HIV risk is ineffective. This finding is consistent with the analysis on the conditions leading to good or bad intuitions [62]. Specifically, the low base rate and the lack of corrective feedback were identified as main conditions leading to bad intuitions, which apply to the present case, i.e., judging HIV risk on person appearance. Thus, even if some cues were statistically associated with actual HIV risk status, reliance on such cues would still provide an ineffective strategy to prevent sexually transmitted diseases. From this perspective, studying how visible cues systematically affect HIV risk ratings may provide the basis for new kinds of public health interventions in which participants can make direct experience with the fallacies of relying on appearance based risk perception to prevent infection with sexually transmitted diseases. Furthermore, targeting intuitive HIV risk perception may also have implications regarding discrimination of certain individuals given the robust literature on stigmatization of HIV positive people [23,63,64].

To relate the current findings to previous research in health psychology and psychophysiology, the present study examined the intuitive perception of HIV risk. However, from a public health perspective it is relevant to determine in future research whether the current findings are specific to HIV or extend to other sexually transmitted diseases. While base rate of HIV is comparably low, chlamydia, gonorrhea, syphilis, and trichomoniasis are STIs with much higher prevalence rates, i.e., an estimated 500 million people becoming infected each year [60], and, similar to HIV, a person can be infected with chlamydia or trichomoniasis without presenting visible symptoms. There is first evidence that the risk stereotype associated with chlamydia is highly similar to the stereotype associated with HIV [65]. Thus, it seems possible that the current findings are not specific to HIV and may generalize to other STIs. However, there is evidence that perceptions of HIV risk can be separated from leukemia risk, which is equally a life-threatening disease with no initial visible signs, but not contagious [52,66,67]. Compared to leukemia, which is viewed as affecting “innocent people by fate”, HIV is, at least on average, associated with more negative attitudes, greater attribution of irresponsibility, and stigma [23,63,64,66]. Interestingly, contrasting HIV and leukemia risk perception revealed a pronounced dissociation in event-related brain potential responses associated with high and low risk for both diseases [52]. This finding implies at least some specificity of intuitive HIV risk perception rather than a generic response to all kinds of diseases. However, a more comprehensive assessment of intuitive risk perception associated with a range of STIs and other diseases is needed to assess the memory representations accessed by intuition for key characteristics of illness representation, i.e., contagiousness and seriousness [67].

The current research is rooted in the health psychological and public health literature on HIV risk behavior in young adults [10,11,68]. However, a highly relevant, but henceforth largely unconnected line of research exists on the topic of face perception in social and evolutionary psychology regarding impressions of health [39], attractiveness [69], and social impressions more broadly [1,70]. We found that HIV risk shows a small to medium association with how “ill-looking” the depicted persons are perceived (*r* = 0.18 for HIV risk to ill-looking), which is substantially lower than e.g. the correlation to responsibility (*r* = 0.6). We interpret this as evidence that people seem to judge HIV risk more based on personality characteristics and less on cues for health. However, several cues that emerged in the cue-utilization analysis, such as ‘reddened eyes’ or ‘spotty skin’, are compatible with cue-utilizations reported for health [35,71,72]. More abstracted cues, such as an ‘exhausted’ or ‘sad’ facial expression were also related to judgments of HIV risk, and such cues have also been reported in the literature on health and disease perception based on facial appearance [72].

The relationship of attractiveness and HIV risk perception is less clear, although there is evidence that physical attractiveness drives sexual interest, which can influence partner selection, and may play a role in the context of HIV risk [45,73,74]. In principle, both, a positive or a negative relationship between attractiveness and HIV risk seem possible: A positive relationship might arise based on the idea that attractive people have more sexual opportunities and thus are likely more at risk. On the other hand, the ‘what-is-beautiful-is-good stereotype’ [75] suggests that more attractive people should have lower risk, and the same prediction arises from the notion that attractiveness ‘advertises’ health [76]. In the current dataset, the correlation between HIV risk and attractiveness is low, and the cue-models trained to predict attractiveness were not able to predict ratings of riskiness, and vice versa. Overall, while the relationship of attractiveness may vary with the attractiveness of the models and experimental methodology [77,78], we observed no robust relationship of attractiveness and perceived HIV risk in our stimulus set.

Future research should examine how these impressions relate to broader notions of disease avoidance [79,80] and to general models of person impressions [1,70]. Over the past years, researchers have made great advancements in modeling facial appearance [81,82] and understanding interactions between cues and higher-order judgments for real-world outcomes [4–6,22,46,72], renewing the interest in the structure of social impressions and their predictability from photographs [83–86]. Recent research suggests a 2- or 3-factor model of social trait impressions [70] comprising approachability/trustworthiness, dominance/masculinity, and youthfulness/attractiveness. Although our work differs with respect to the stimuli used (faces vs. more naturalistic photographs of persons) as well as regarding the concrete traits studied, previous findings using similar factor-analytic techniques found a comparable organization in which a factor comprising trustworthiness, responsibility, and risk was dissociable from a factor for attractiveness, health, and willingness to interact [27]. To comprehensively position “HIV risk” within this social trait space requires studying a broader variety of images and the assessment of more traits [83]. Interestingly, in the domain of trust, recent research demonstrated that different images of the same person are associated with different ratings of trust [87]. Presenting multiple images of the same person in future research may not only provide further insight into the association of risk perception with the key characteristics of the HIV risk stereotype but would also reveal the extent to which risk perception varies with situational context.

The present study has limitations with regard to the stimulus materials as well as the examined cues and person characteristics. While our stimulus materials were selected to represent naturalistic person presentations on social media, a larger number of stimuli would be desirable. Related to the issue of stimulus sampling, future work should extend our findings to different cultural contexts, and vary both the observers and targets. Extending our work beyond the ‘western’ setting in which our study was conducted could provide insights into commonalities and differences of social inferences across cultures [88]. Additionally, in recent work we found differences in HIV risk ratings based on both the gender of the person that is to be judged as well as the raters’ gender [30]. Within the current work, the entire cue-criterion-correlation vector for males and females showed a large correlation (*r* = 0.72, *p* < 0.001). Future work might examine possible gender commonalities and differences systematically using larger sample sizes. Furthermore, while we undertook considerable effort to include relevant cues to person perception based on literature research and focus group discussions, it is always possible that the inclusion of additional cues would result in larger cue-utilization coefficients. However, the strong association between riskiness and multiple cues may be considered as evidence that our cue selection covered most obvious aspects. We also note that the ratings of HIV risk as well as other characteristics are inherently relative and should be interpreted with respect to the current set of stimuli and questions. Furthermore, it would be helpful to see how HIV risk impressions relate to additional person characteristics, including for instance competence, warmth, or intelligence [1,89]. Additionally, assessing measures of confidence in the perceived person characteristic may provide valuable information on the association between trust, responsibility, and HIV risk ratings. Such work may also point to subtle differences when moving from general to more specific person characteristics.

### Summary and conclusion

This study reveals visual cues that are systematically linked to snap judgments about a person’s HIV risk. HIV risk impressions appear to be embedded in a stereotypical set of beliefs about negative personality attributes. Knowing only a handful of cues is sufficient to predict perceptions of HIV risk. These findings provide insight into the phenomenon of intuitive risk perception and can be used to design health campaigns and interventions aimed at reducing the burden of HIV and sexually transmitted infections.

## Supporting information

S1 Table ICCs for all cues. (DOCX)
