# Supplementary material for: Visual cues that predict intuitive risk perception in the case of HIV
Source: PLoS One. 2019 Feb 20;14(2):e0211770. doi: 10.1371/journal.pone.0211770 (PMC6382111; doi:10.1371/journal.pone.0211770)
Supplement: S1 Table — (DOCX) [file pone.0211770.s001.docx]

**Table S1. ICCs for all cues.**

| **CUE** | **ICC** |
| --- | --- |
| **Face: Eyes** |  |
| Dark (vs. no dark rings under eyes) | 0.71 |
| Reddened (vs. no reddened eyes) | 0.54 |
| Dark (vs. bright eyes) | 0.85 |
| **Face: View** |  |
| Coquettish (vs. no coquettish gaze) | 0.82 |
| Averted (vs. front facing gaze) | 0.97 |
| Tired (vs. alert gaze) | 0.84 |
| **Face: Hair** |  |
| Ungroomed (vs. groomed hair) | 0.78 |
| Long (vs. short hair) | 0.93 |
| Fashionable (vs. unfashionable hairstyle) | 0.80 |
| Dark (vs. bright hair) | 0.95 |
| **Face: Mouth** |  |
| Smile (vs. non smile ) | 0.90 |
| Yellow (vs. white teeth) | 0.81 |
| Full (vs. narrow lips) | 0.81 |
| **Face: Skin** |  |
| Unhealthy (vs. healthy skin) | 0.78 |
| Pale (vs. tanned skin) | 0.62 |
| Pimply (vs. pimple free skin) | 0.75 |
| Many (vs. few skin folds) | 0.70 |
| Spotty (vs. spot free skin) | 0.77 |
| Greasy (vs. dry skin) | 0.68 |
| Lots of (vs. no skin visible) | 0.93 |
| **Facial Configuration** |  |
| Babyish (vs. mature Face) | 0.77 |
| Feminine (vs. masculine face) | 0.76 |
| Round (vs. narrow face) | 0.90 |
| Worn (vs. fresh face) | 0.69 |
| Ugly (vs. beautiful face) | 0.83 |
| Red (vs. pale cheeks) | 0.86 |
| Narrow (vs. full jaws) | 0.71 |
| Average (vs. unusual face) | 0.63 |
| Reddened (vs. no reddened face) | 0.82 |
| Symmetric (vs. unsymmetric face) | 0.54 |
| **Facial Expression** |  |
| Happy (vs. sad expression) | 0.94 |
| Exhausted (vs. powerful expression) | 0.75 |
| Worried (vs. unworried expression) | 0.88 |
| Serious (vs. blithely expression) | 0.92 |
| Angry (vs. cheerful expression) | 0.88 |
| Friendly (vs. grumpy expression) | 0.93 |
| **Body: Figure** |  |
| Musculous (vs. not musculous stature) | 0.74 |
| Overweight (vs. underweight ) | 0.92 |
| Well (vs. badly proportioned Stature) | 0.87 |
| Tall (vs. low height) | 0.70 |
| Tense (vs. relaxed posture) | 0.75 |
| **Body: Appearance** |  |
| Ungroomed (vs. groomed appearance) | 0.84 |
| Lot of (vs. no body adornment) | 0.82 |
| Worn out (vs. intact clothes) | 0.71 |
| Provocative (vs. reserved clothes) | 0.86 |
| Unconventional (vs. conventional appearance) | 0.47 |
| Fashionable (vs. unfashionable appearance) | 0.85 |
| Dark (vs. bright clothes) | 0.94 |
| Clean (vs. dirty clothes) | 0.79 |
| **Setting** |  |
| Pallid (vs. colorful background) | 0.90 |
| Unorganized (vs. organized background) | 0.80 |
| Alcohol (vs. no alcohol visible) | 0.96 |
| Picture taken inside (vs. outside.) | 0.97 |
| Picture taken in nature (vs. civilization.) | 0.95 |
| Cigarettes (vs. no cigarettes visible) | 0.86 |
| Food (vs. no food visible) | 0.88 |
| Dark (vs. well lit background) | 0.91 |
| During sports activities (vs. not.) | 0.76 |
